# Can CFD establish a connection to a milder COVID-19 disease in younger people? Aerosol deposition in lungs of different age groups based on Lagrangian particle tracking in turbulent flow

**DOI:** 10.1007/s00466-021-01988-5

**Published:** 2021-03-19

**Authors:** Jana Wedel, Paul Steinmann, Mitja Štrakl, Matjaž Hriberšek, Jure Ravnik

**Affiliations:** 1 Institute of Applied Mechanics, University of Erlangen-Nuremberg, Erlangen, Germany; 2 Glasgow Computational Engineering Center, University of Glasgow, Glasgow, Scotland; 3 Faculty of Mechanical Engineering, University of Maribor, Maribor, Slovenia

**Keywords:** SARS-CoV-2, Aerosol, CFD, OpenFOAM

## Abstract

To respond to the ongoing pandemic of SARS-CoV-2, this contribution deals with recently highlighted COVID-19 transmission through respiratory droplets in form of aerosols. Unlike other recent studies that focused on airborne transmission routes, this work addresses aerosol transport and deposition in a human respiratory tract. The contribution therefore conducts a computational study of aerosol deposition in digital replicas of human airways, which include the oral cavity, larynx and tracheobronchial airways down to the 12th generation of branching. Breathing through the oral cavity allows the air with aerosols to directly impact the larynx and tracheobronchial airways and can be viewed as one of the worst cases in terms of inhalation rate and aerosol load. The implemented computational model is based on Lagrangian particle tracking in Reynolds-Averaged Navier–Stokes resolved turbulent flow. Within this framework, the effects of different flow rates, particle diameters and lung sizes are investigated to enable new insights into local particle deposition behavior and therefore virus loads among selected age groups. We identify a signicant increase of aerosol deposition in the upper airways and thus a strong reduction of virus load in the lower airways for younger individuals. Based on our findings, we propose a possible relation between the younger age related fluid mechanical protection of the lower lung regions due to the airway size and a reduced risk of developing a severe respiratory illness originating from COVID-19 airborne transmission.

## Introduction

Flows with dispersed particles are of great interest, since they can be found in numerous fields of engineering and medical science. In this context, the ongoing COVID-19 pandemic can be considered as a recent topic. The causative agent of the disease is known as SARS-CoV-2 that primarily targets the respiratory tract and can manifest mostly as pneumonia when affecting the lower respiratory tract and less likely as acute respiratory distress syndrome (ARDS) [13, 32]. The virus is known to be transmissible via contacts and droplets as well as aerosols [32, 38]. Duguid [10] showed that by a single sneeze, infected hosts can easily generate up to a few million contaminated droplets and aerosols. In many studies aerosols are defined as particles with a diameter $d_p\le 5\,\upmu$ m and larger particles as droplets [11, 36]. However, there have been some suggestions that widen that range and postulate particles up to an aerodynamic diameter of $10{-}20\,\upmu$ m as aerosols, due to their ability to linger prolongedly in the air and reach deeper in the lung, [7]. Thomas et al. [33] stated that larger particles mostly deposited in the upper airways whereas small aerosols are more prone to bypass the mechanical defense mechanisms and penetrate deeper into the lung, causing typical disease profiles related to the alveolar region. After the host released the contaminated aerosols, the SARS-CoV-2 virus has been found to remain viable for $3\,$h, increasing the risk for airborne spread of COVID-19 [7]. On November 8th, 2020, approximately one year after the outbreak, COVID-19 affects a significant proportion of people with 49.7 million reported cases and 1.2 million deaths worldwide [37]. However, the SARS-COV-2 virus is not yet fully understood and there are still high uncertainties remaining. This involves the size distribution of droplets and aerosols, the aerosol viral load and the minimum number of inhaled SARS-CoV-2 viruses that are required to infect an individual [26]. Moreover, it is still unknown why there is an unequal distribution and course of infections among the population as it is less frequently diagnosed among children [6]. As mentioned by Thomas et al. [33], the biometry and therefore deposition profiles are affected by factors like age, body weight, breathing mode, gender and health state. This leads to the question, whether pure mechanical effects, like biometry and breathing mode of children, render a decisive difference in aerosol deposition behavior and consequently virus load compared to grown ups.

Given the fact that in vivo and in vitro experiments are mainly limited due to human safety and image resolution [19], computational fluid dynamics (CFD) can play an important role to enable new insights in this field. In this paper we first validate our numerical lung model setup by comparison with the in vitro and in silico benchmark case of Koullapsis et al.[18]. Moreover, we discuss the effects of age-related lung sizes on the deposition of cough generated aerosols and therefore virus doses in human airways. This is chosen to investigate the correlation of age and regional aerosol deposition accessing the SARS-CoV-2 pandemic from a fluid mechanical point of view. The general purpose of this paper is to provide new insights in age-related aerosol deposition.

The paper is organized as follows: In Sect. 2, the governing equations are reviewed. Moreover, the lung model is validated with respect to benchmark models for the case of a human lung replica in Sect. 3. Additionally, Sect. 4 contains the setup of the present lung sizes to model aerosol deposition in various human age-groups as well as results and discussion of local aerosol load in different airway sizes. Finally, Sect. 5 summarizes the paper and presents the main conclusions.

## Methods

### Airway geometry

**Fig. 1:**
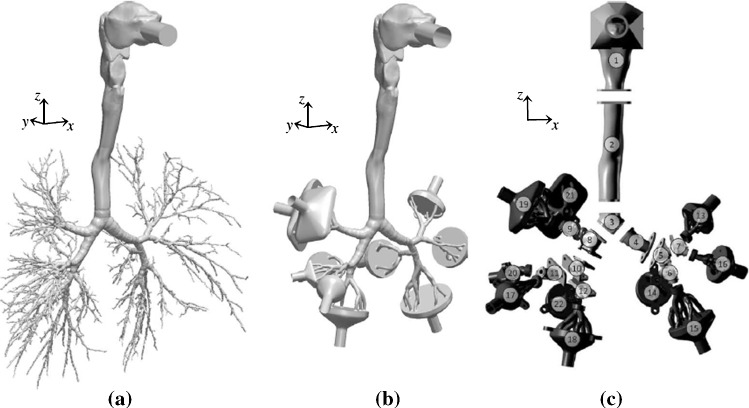
**a** Original airway, **b** benchmark case, **c** segmented model [18]

There is a variety of airway geometries that is used in CFD. Early models employed simplified artificial geometries mainly basing on the symmetric model of Weibel et al. [34] In recent years, medical imaging enabled a detailed view of the human airway and provided more realistic replicas [19]. The geometry considered in this paper, see Fig. 1b, is the same as in the benchmark case of Koullapsis et al. [18] and is originally used in [3, 4, 15, 23]. The benchmark case consists of a simplified lung model that was adopted to measure the regional deposition ratios. The realistic airway geometry and the simplified airway model used in the benchmark case are shown in Fig. 1. More details related to the lung model generation can be reviewed in [18].

### Flow field

We employ the Reynolds Averaged Navier Stokes (RANS) equations using the k-$\omega$-SST model in order to study the steady state flow in the replica of the human lung. The governing RANS equations for an incompressible fluid, which are solved using OpenFOAM$^{\circledR }$ [28, 35], are given by

1 $$\begin{aligned}&\mathrm{div}\bar{\mathbf {u}}=0\,, \end{aligned}$$

2 $$\begin{aligned}&\mathrm{d_t}(\rho \bar{\mathbf {u}})+\mathrm{div}(\rho \bar{\mathbf {u}}\otimes \bar{\mathbf {u}} + \varvec{\tau }^{\mathrm{RANS}} )= -\mathrm{grad}\bar{p} \nonumber \\&\quad +\, \mathrm{div}\bar{\varvec{\tau }} +\bar{\mathbf {f}}_D\,, \end{aligned}$$

with

3 $$\begin{aligned} \bar{\varvec{\tau }}:=\mu \,\mathrm{grad}^{\mathrm{SYM}} \bar{\mathbf {u}}\,, \end{aligned}$$

representing the mean viscous stress tensor components and

4 $$\begin{aligned} \varvec{\tau }^{\mathrm{RANS}}:=\rho \bar{\mathbf {u}}_i' \otimes \bar{\mathbf {u}}_j'\,, \end{aligned}$$

denoting the Reynolds stresses [12]. Here $\bar{\mathbf {u}}$ and $\bar{p}$ describe the Reynolds-averaged fluid velocity components and pressure and $\rho$ denotes the fluid density. Moreover, the $'$-sign represents fluctuations. Additional body forces can be found in $\bar{\mathbf {f}}_D$. The closure problem to constitutively express $\varvec{\tau }^{\mathrm{RANS}}$ can be solved by employing approximate turbulence models like the k-$\omega$-SST model [12]. OpenFOAM$^{\circledR }$ uses the finite volume method to discretise the above equations.

### Particles

Dispersed flows can be captured by the Lagrangian-Eulerian approach. Hence, the Navier-Stokes equations are solved for the continuous phase (air) as mentioned in Sect. 2.2 and the motion of particles (aerosols) in fluid is described in a Lagrangian manner [14]. To this end, a set of ordinary differential equations is evaluated along the particle trajectory to obtain the change of particle location and motion. These equations are given by Newton’s second law and render for a spherical particle point mass:

5 $$\begin{aligned}&\mathrm{D_t}\mathbf {x}_p:=\frac{d \mathbf {x}_p}{dt}=\mathbf {u}_p, \end{aligned}$$

6 $$\begin{aligned}&\mathrm{D_t} (m_p \mathbf {u}_p ):= m_p \frac{d\mathbf {u}_p}{dt}=\sum \mathbf {F}_i, \end{aligned}$$

where $\mathbf {x}_p$ is the particle’s position vector, $\mathbf {u}_p$ the particle’s velocity, $m_p=\rho _p d_p^3\pi / 6$ the mass of the spherical particle and $\sum \mathbf {F}_i$ represents the sum of forces acting on the particle. [14] In this study, the aerosol dimensions are estimated to be at the scale of $1 \le d_p\le 10~\upmu$ m. Hence, the forces containing the major influence on the particle trajectory are the drag force $\mathbf {F}_D$, the buoyancy force $\mathbf {F}_B$ and the gravitational force $\mathbf {F}_G$. Other forces like Brownian motion, added mass, and Basset history force are considered as negligible [14]. Thus, the force balance equation simplifies to:

7 $$\begin{aligned} m_p \frac{d \mathbf {u}_p}{d t} = \mathbf {F}_D + \mathbf {g} V_p\left[ \rho _p-\rho _f\right] \,, \end{aligned}$$

where $m_p$, $V_p$, $\rho _p$ are the mass, volume and density of the particle, respectively, $\rho _f$ denotes the fluid density and $\mathbf {g}$ is the gravitational acceleration. The drag force for spherical particles in OpenFOAM$^{\circledR }$ is implemented as follows: [14]

8 $$\begin{aligned} \mathbf {F}_D = \frac{3}{4} \frac{\rho _f}{\rho _p} \frac{m_p}{d_p} C_D [\mathbf {u} -\mathbf {u}_p]|\mathbf {u} -\mathbf {u}_p|, \end{aligned}$$

where $d_p$ is the particle diameter and $C_D$ is the drag coefficient. OpenFOAM$^{\circledR }$ uses the following empirical relation for the drag coefficient [14]:

9 $$\begin{aligned} C_D:= {\left\{ \begin{array}{ll} \frac{24}{\mathrm{Re}_r}[1+\mathrm{Re}_r^{2/3}/6]; &{} \mathrm{Re}_r\le 1000\,.\\ 0.424;&{} \mathrm{Re}_r\ge 1000\,. \end{array}\right. } \end{aligned}$$

where $\mathrm{Re}_r:=\rho _f d_p |\mathbf {u_p} -\mathbf {u}|/\mu$ denotes the particle Reynolds number based on the relative velocity [8]. The buoyancy and gravitational force are usually combined and computed jointly as [17]:

10 $$\begin{aligned} \mathbf {F}_B+\mathbf {F}_G=m_p\mathbf {g} \left[ 1-\frac{\rho _f}{\rho _p}\right] =\mathbf {g} V_p\left[ \rho _p-\rho _f\right] \,. \end{aligned}$$

The equations are solved with the *icoUncoupledKinematicParcelFoam*^1^ solver of OpenFOAM$^{\circledR }$.

**Table 1:** Computational details of present model and benchmark simulations (LES1, RANS1) of [18]

	LES1	RANS1	PRESENT
Flow solver:	OpenFOAM$^{\circledR }$	OpenFOAM$^{\circledR }$	OpenFOAM$^{\circledR }$
Turbulence model:	LES	RANS	RANS
	Dynamic Smagorinsky [20]	k-$\omega$-SST [25]	k-$\omega$-SST [25]
Inlet b.c.:			
*P*:	Atmospheric	Atmospheric	Atmospheric
*U*:	Turbulent	Turbulent inlet	Flowrate$^{\mathrm{a}}$ /
	(Mapped inlet)		Parabolic velocity$^{\mathrm{b}}$
Outlet b.c.:			
*P*:	Zero-gradient	Zero-gradient	Zero-gradient
*U*:	Specified flowrates	Specified flowrates	Specified flowrates

$^{\mathrm{a}}$(Mesh 1), $^{\mathrm{b}}$ parabolic profile: nth power law with n=7 (Mesh 2)

**Table 2:** Mesh statistics of present model and benchmark simulations (LES1, RANS1) of [18]

	LES1	RANS1	PRESENT (Mesh 1)$^{\mathrm{a}}$	PRESENT (Mesh 2)$^{\mathrm{a}}$
Cells	50 M	12 M	6 M	20 M
Boundary layers	3–5	0	3	3

$^{\mathrm{a}}$Near wall distance: $y^+\approx 1$

### Turbulent dispersion model

To account for the interaction of the particles with the turbulent eddies, the instantaneous velocity $\mathbf {u}=\bar{\mathbf {u}}+ \mathbf {u}'$ of the fluid is required. As this field is not accessible from the RANS equations and only the averaged velocity $\bar{\mathbf {u}}$ is available, additional models are required to properly estimate the fluctuation velocity. Here we employ the OpenFOAM$^{\circledR }$ model *StochasticDispersionRAS* [16] where $\mathbf {u}'$ is computed to disturb the velocity field in a random direction, with a Gaussian distribution of zero mean and variance $\sigma$[18]. It relates the velocity fluctuations to the turbulent kinetic energy *k* as follows

11 $$\begin{aligned} \mathbf {u}'=\xi \mathbf {d} \sqrt{\frac{2}{3}k}\,, \end{aligned}$$

where $\mathbf {d}$ is an additional random vector, $\xi$ denotes random numbers with zero mean and unit variance of Gaussian distribution and *k* is the turbulent kinetic energy [14]. The model assumes isotropic turbulence, rendering the standard deviation $\sigma$ as

12 $$\begin{aligned} \sigma =\sqrt{\frac{2}{3}k}=\sqrt{u_{1}'^2}=\sqrt{u_{2}'^2}=\sqrt{u_{3}'^2}\,, \end{aligned}$$

with $u_{1}$, $u_{2}$, $u_{3}$ describing the velocity coefficients in Cartesian coordinates [14, 16].

### Limitations

To model the flow of aerosols, we restricted ourselves in the present study to the following conditions:

- dilute flow allowing for one-way coupling of particles and fluid,
- assumption of isotropic turbulence,
- steady state flow field.

In addition, the size of the aerosols under investigation ($1 \le d_p \le 10\,\upmu$ m) is sufficiently small, so their surface tension is strong enough to solely behave like small spherical rigid particles [2].

## Numerical verification of the lung model

A direct validation of the present model by comparison with a direct numerical simulation (DNS) is difficult, due to the complex geometry of the human airway. Nevertheless, the present lung model can be validated indirectly by comparing it with the benchmark results of Koullapsis et al. [18].

### Numerical setup

The simplified airway model used in the benchmark case is shown in Fig. 1b. The benchmark analysis consists of an in vitro experiment and five different in silico approaches. To evaluate the results of the present model, the benchmark LES1 simulation was selected as the main reference as it showed good agreement with the in vitro results [18]. Furthermore the benchmark study demonstrated that the RANS1 result was the most accurate RANS simulation compared to RANS2 and RANS3. Hence, both LES1 and RANS1 are used for comparison with the present model. Their numerical setup is provided in Table 1. In addition, the mesh statistics are presented in Table 2.

**Fig. 2:**
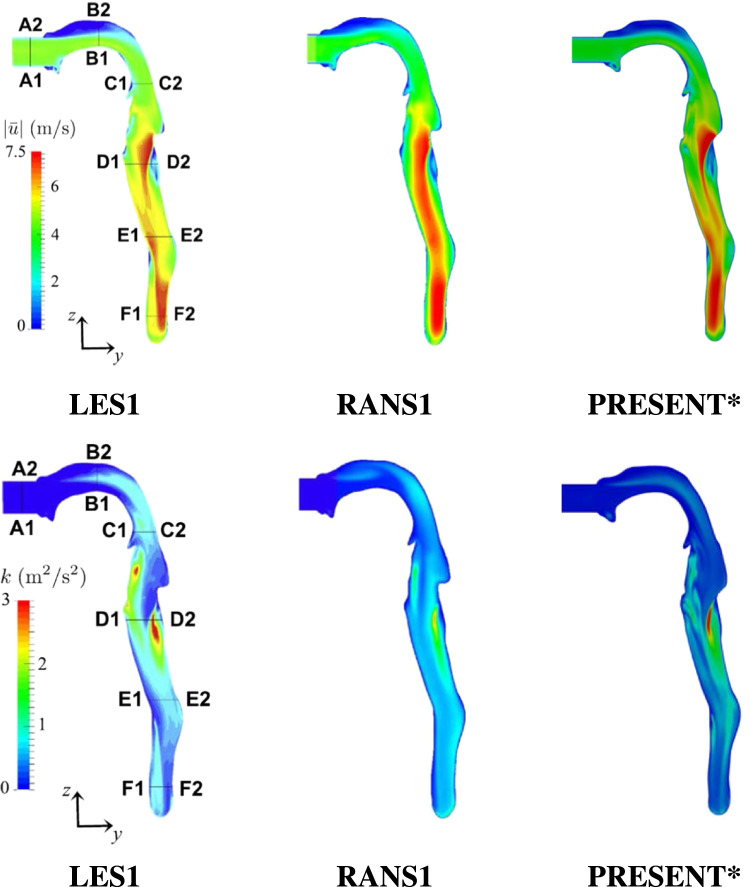
Velocity profile and turbulent kinetic energy in the central sagittal plane. Reference data of LES1 and RANS1 from [18], *(Mesh 2)

### Flow field

In Fig. 2 the contours of mean velocity magnitude and turbulent kinetic energy are compared in the central sagittal plane of the airways mouth-throat region. It is evident that a good qualitative agreement of the mean velocity distribution is achieved for the present mode (Mesh 2) compared to the LES1. The Y-shaped velocity pattern of the LES1 simulation can be resolved with the present model (Mesh 2) approach, whereas it is absent in the RANS1 velocity field. The level of turbulent kinetic energy for both RANS cases is lower in comparison to the LES1 results. However, the present model (Mesh 2) is able to capture the main characteristics e.g. maximum of the turbulent kinetic energy in the throat region close to the (D1–D2) cross-section. The discrepancies between RANS methods are likely due to the lower grid resolution of the RANS1 model and the absence of boundary layers.

**Fig. 3:**
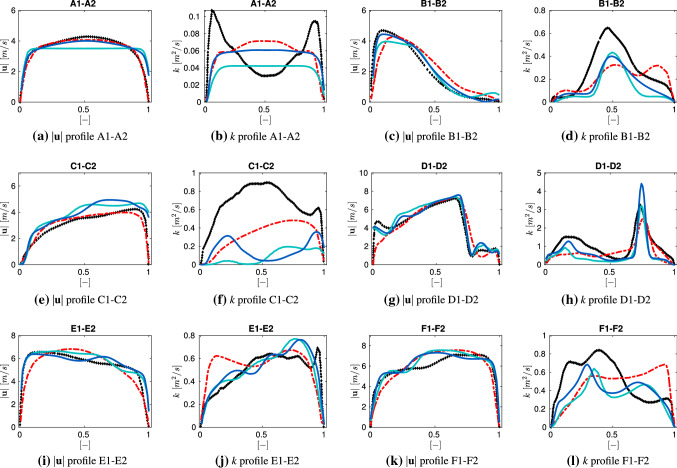
Velocity ($|\mathbf {u}|$) and turbulent kinetic energy (*k*) cross-sections (60 l/min);   LES1,   RANS1,  PRESENT (Mesh 1),  PRESENT (Mesh 2). (Color figure online)

In the next step a more detailed analysis of the velocity and kinematic turbulent energy fields is conducted by comparing the respective profiles at selected cross-sections. The results are displayed in Fig. 3. Here also the PRESENT (Mesh 1) cross-sections are included, which were evaluated on a significantly coarser grid. The location of the cross-sections is highlighted in Fig. 2, with exact locations estimated from Koullapsis et al. [18]. In the (A1–A2) section, which represents the inlet region, the turbulent velocity profile can be captured by all numerical setups, except the PRESENT (Mesh1) setting that employed a fixed inlet flow rate. Moreover, the typically low velocity profiles in the low mouth and pharynx region, depicted in (B1–B2) and (C1–C2) respectively, can be reproduced by all methods. In addition, the present models are able to capture the effects in the acceleration region (D1–D2) of the pharynx. Besides, the (E1–E2) (F1–F2) cross-sections of the present model achieve good agreement with the LES1 and RANS1 reference results. Even though the exact locations of the reference profiles were not provided in [18], the mean velocity profiles achieved low discrepancies compared to the LES1 and RANS1 cross-sections. In the case of turbulent kinetic energy, the deviation between the present models and the LES1 results slightly increases and the RANS results lead, mainly in the upper mouth-throat region (B1–B2) (C1–C2), to an under-prediction. As observed by Koullapsis et al. [18], larger discrepancies in the profiles of the turbulent kinetic energy occur in the upper regions of the airway across simulations. This deviation can partially be linked to different inlet conditions as well as mesh resolution among LES1 and RANS models. A further cause is the pronounced anisotropic nature of the flow in this region, a consequence of some significant changes in the airway’s geometry. Capturing the anisotropy effects is a known deficiency of the RANS based model, which can significantly be improved by applying LES models, however at a much higher computational cost. Since the k-$\omega$-SST model still performed relatively well compared to the high-end LES validation case, capturing the main characteristics of the turbulent kinetic energy, its reasonable computational cost led to its choice in the framework of the targeted parametric analysis. Additionally, it is indicated that the impact of the different inlet conditions of PRESENT (Mesh 1) on the turbulence kinetic energy as well as the velocity, is low downstream of the mouth region.

### Verification of particle deposition

**Fig. 4:**
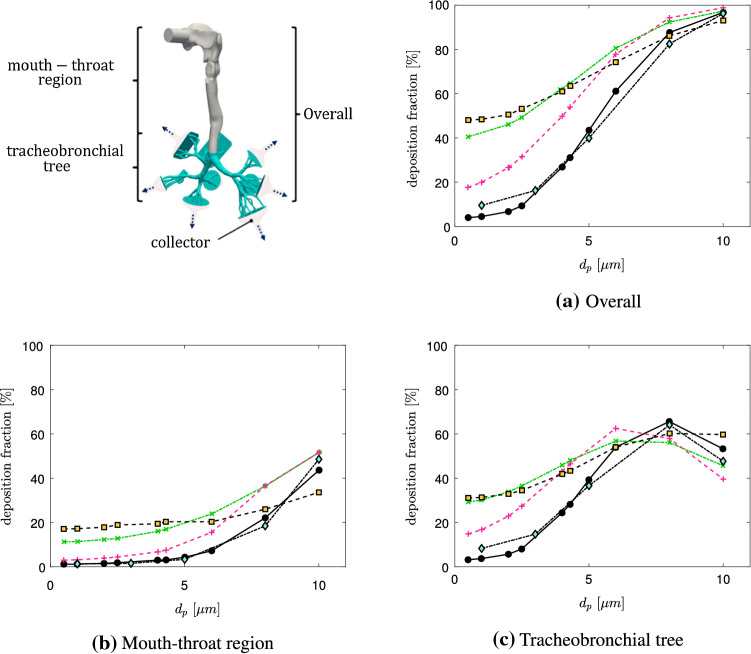
Particle deposition (60 l/min);  LES1,  LES2,  LES3,  RANS1,  PRESENT (Mesh 2). (Color figure online)

To verify the particle deposition behavior, the present lung model is compared to the LES1 and RANS1 deposition fractions of Koullapsis et al. [18] for three different flow rates and a diameter range of $1\le d_p\le 10\,\upmu$ m. According to Koullapsis et al. [18], the particles are considered to be diethylhexyl sebacate particles in ambient air temperature ($\rho _p=914\,\mathrm{kg/m}^3$) and are distributed uniformly across the inlet. Details of the particle-insertion are presented in Table 3. A thorough description of the reference simulations that are compared in Fig. 4 is provided in [18].

In the following, the human airway is subdivided in three different regions of interest. These are the mouth-throat, tracheobronchial tree as well as a combination of both, which is referred to as the overall region. The mouth-throat region includes the oral-cavity and trachea. The tracheobronchial tree describes the human lung more downstream, excluding the collectors, which represent the lower airway regions. Figure 4 displays the deposition behavior of the selected particle range with an inhalation of $60~\mathrm{l/min}$ in the mouth-throat, tracheobronchial tree and overall lung region.

**Table 3:** Computational details of particle tracking for verification and reference simulations [18]

	LES1	RANS1	PRESENT
Time integration scheme	Implicit Euler	Implicit Euler	Implicit Euler
Forces on particles	Drag$^{\mathrm{a}}$, gravity, brownian	Drag$^{\mathrm{a}}$, gravity	Drag$^{\mathrm{a}}$, gravity
Wall interaction	Stick	Stick	Stick
Cunningham correction ($C_c$)	Yes	Yes	–
Turbulent dispersion	−	Continuous	Continuous
	–	Random walk	Random walk
Number of particles	100,000	100,000	100,000

$$^{\mathrm{a}}$$Drag coefficient ($$C_D$$) [31]

**Fig. 5:**
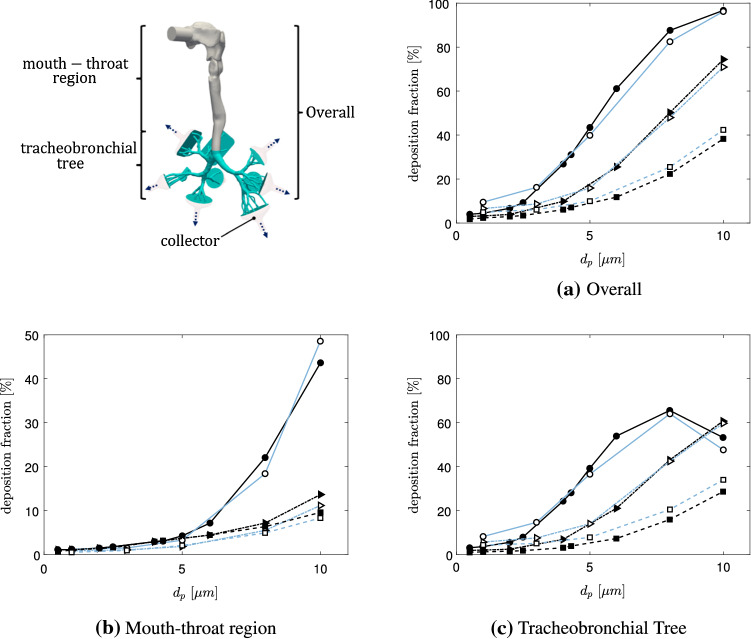
Particle deposition for different flowrates;  LES1-60 l/min,  PRESENT-60 l/min,  LES1-30 l/min,  PRESENT-30 l/min,  LES1-15 l/min,  PRESENT-15 l/min

A wide range of results is generated by employing the different methods (LES1-3, RANS1-3) in the reference [18]. It is obvious that the deviation between the present model (PRESENT (Mesh 2)) and the benchmark LES1 results in the overall airway geometry is small, compared to the addition provided method. Moreover, it is shown that the numerical results in the mouth-throat as well as the tracheobronchial tree are respectively in a good agreement, proving the validity of the present model. However, in the tracheobronchial region, the deviation increases by decreasing the particle diameter. In the mouth-throat region, in contrary, the difference between the present model and the reference vanishes towards smaller particles. In Fig. 5 the LES1 deposition fractions of the benchmark case and the present model are compared regarding three different flow rates (60, 30 and $15\,\mathrm{l/min}$). For all three flow rates an over-prediction of deposition occurs for smaller particles in the tracheobronchial tree compared to the LES1 reference results. The differences in the mouth-throat region are very small for all flow rates. Overall the model is able to reproduce the LES1 results in a sufficient way, rendering the model suitable to be employed for further investigations.

## Aerosol deposition in lungs of different age groups

In this section the verified lung model is further employed to account for aerosol deposition in different lung sizes. The main interest is to evaluate possible variations in aerosol deposition of different particle sizes in human airways among children and grown-ups. The following age groups are considered: toddler (1–3 years), preschooler (3–6 years), school-age (6–12 years), adolescent (12–18 years) and adult ($\ge$18) [27].

### Model generation

Due to the limited availability for sufficient lung models of different age groups, the airway geometries of children are generated by scaling the adult lung volume of [18]. The scaling factor is set to represent the ratio of the total volume capacity (TLC) of the considered age group compared to the TLC of a grown-up. The TLC describes the ’maximum volume of air that the lungs can hold after a maximum inspiration’[1]. For adults, the TLC is taken to be $5.7\,\mathrm{l}$ as mentioned by Aung et al. [1]. To estimate the TLC of children, the relationship proposed by Lyons et al. [24] is considered:

13 $$\begin{aligned} TLC=30.71\times H+29.35\times W-2545, \end{aligned}$$

where *H* is the height in cm and W is the mass in kg. The flow rates are estimated by taking the average breathing frequency of the considered age groups times the tidal volume (TV). The TV describes the ’volume of air that is breathed in and out in a single quiet breath’[1]. The average TV of a resting male adult is estimated as $630\,\mathrm{ml}$ [30]. To obtain the TV of children the following estimation for mechanical ventilation is employed [9]:

14 $$\begin{aligned} TV= W \times 6\,\mathrm{ml/kg}. \end{aligned}$$

The studied characteristic children, which represent the average weight and height of the given ages, are provided in Table 4. In the present study, the set-up of the simulation is the same as in Sect. 3.1. In Addition, the computational domain is scaled by the TLC ratio.

**Table 4:** Description of age groups

Name	Group	Age [years]	PRR.$^{\mathrm{a}}$	Weight [kg]	Height [m]
Child (Age 1)	Infant	1–2	24–40	12.0	0.84
Child (Age 3)	Toddler	3–4	24–40	16.5	1.01
Child (Age 5)	Pre-schooler	5–6	22–34	21.0	1.15
Child (Age 7)	School-age	7–8	18–30	26.9	1.28
Child (Age 9)	School-age	9–10	18–30	34.3	1.39
Child (Age 13)	Adolescent	13–14	12–16	53.9	1.64
Adult (Male)	Adult	$\ge ~$18	12	–	–

$^{\mathrm{a}}$Pediatric Respiratory Rate [breaths/min] [27]

**Fig. 6:**
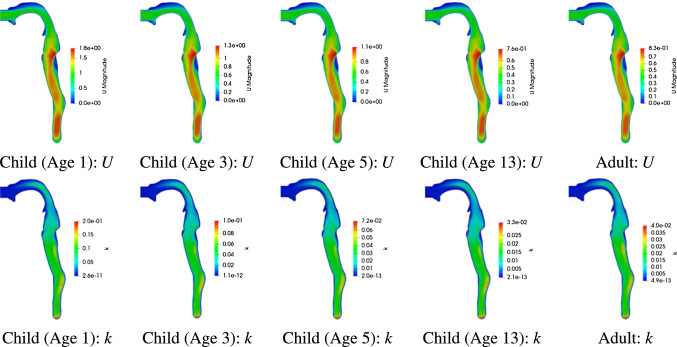
Velocity profile *U* and turbulent kinetic energy *k* in the central sagittal plane for various age-groups

For the youngest child (Age 1) we estimate a mean inlet velocity of $\bar{U}_{inlet}=0.74\,\mathrm{m/s}$ whereas $\bar{U}_{inlet}=0.33\,\mathrm{m/s}$ is obtained for the male adult. Considering the different inlet areas, we achieve an average (inlet) Reynolds number of $Re = 392$ for the child (Age 1) and $Re = 436$ for the adult. Therefore, the flow characteristics are approximately similar across the simulations. The flow field and turbulent kinetic energy in the central sagittal plane are displayed among various age-groups in Fig. 6. Observe the different scaling of these quantities in Fig. 6 despite otherwise similar patterns of *U* and *k*.

### Modeling of aerosol

The particles are considered to be cough-generated aerosols with a density of $\rho _p=1704\,\mathrm{kg/m}^3$ as proposed by [21]. Moreover, a quantity of 100, 000 aerosols are distributed randomly at the inlet and released over a time span of $5\,\upmu$ s. Furthermore, the initial parcel/particle velocity ($U_0$) is set to zero. Besides, the considered aerosol range is $1-10\,\upmu$ m as discussed in Sect. 1. In addition, the aerosols are assumed to stick to the airway wall once a particle comes into contact with this boundary, to mimic the mucus layer on the inner walls of the airways [18]. Furthermore, the particle tracking time step was set to $10\,\upmu$ s for the male adult and children (Age 13–5) as well as $5\,\upmu$ s for child models (Age 3–1), ensuring a maximal particle Courant number of $Co_p\le 1.0$.

### Results and discussion

#### Influence of lung size on aerosol deposition

**Fig. 7:**
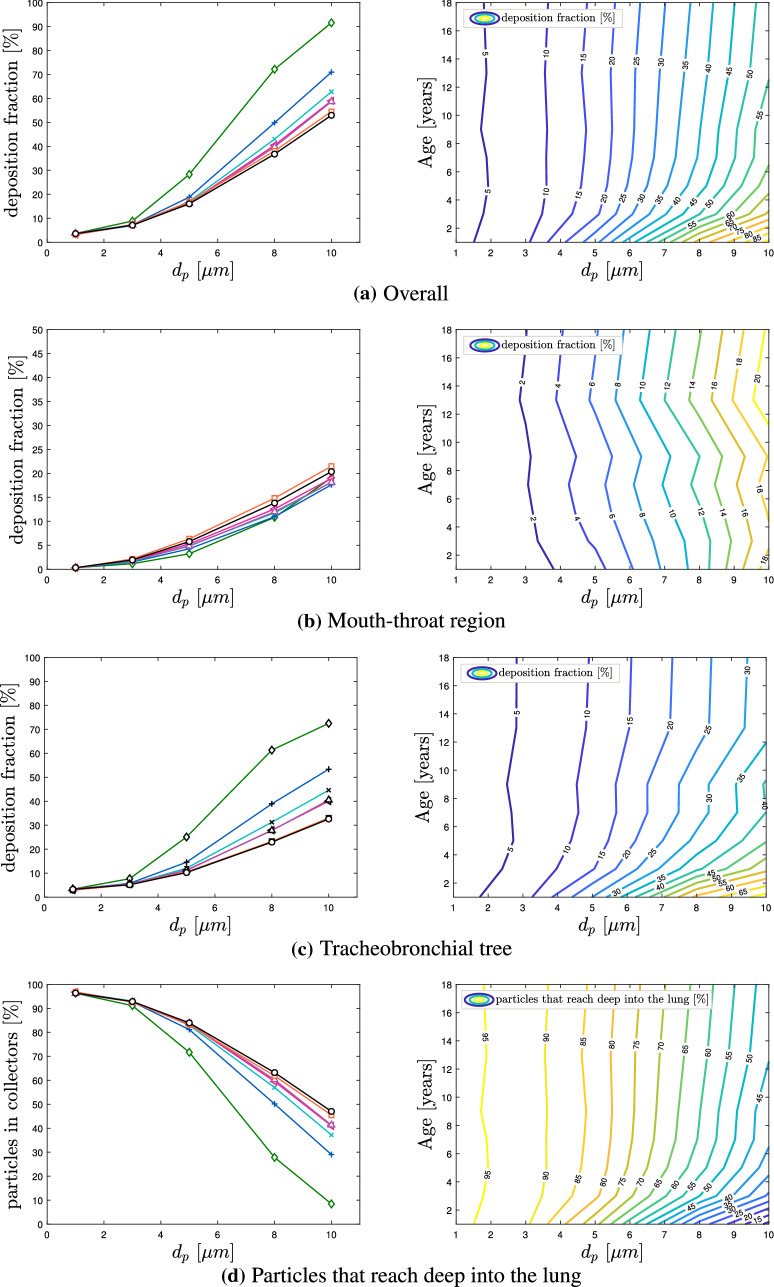
Aerosol deposition for different lung sizes;  Child (Age 1),  Child (Age 3),  Child (Age 5),  Child (Age 7),  Child (Age 9),  Child (Age 13),  Adult (Male). (Color figure online)

Firstly, after $2.5\,$s the deposition fractions of the mouth-throat, the tracheobronchial tree and the cumulative results (overall) were investigated. Figure 7a–c displays the aerosol deposition fractions over particle size for seven different lung volumes. The cumulative results, displayed in Fig. 7a, show a clear trend for all considered lung sizes. The deposition fraction of all artificial individuals grows with increasing aerosol diameter. However, Fig. 7b indicates a more moderate increase in the mouth-throat region. Therefore, the main impact of the particle diameter on the aerosol deposition occurs in the tracheobronchial tree, which is presented in Fig. 7c.

The effect of particle size influence coincides with Sect. 3.3. Additionally, aerosols with a diameter of $d_p=1\,\upmu$ m behave similarly for all lung dimensions and reach an identical deposition fraction in the mouth-throat as well as in the tracheobronchial tree. Consequently, these aerosols experience the lowest deposition rate, indicating that aerosols with $d_p=1\,\upmu$ m are more prone to travel deeper into the lung. However, Fig. 7a, c, indicate that the aerosol deposition fraction varies significantly among the considered age groups for particles with $d_p>3\,\upmu$ m. In the mouth-throat region, presented in Fig. 7b, the aerosol deposition is mainly unaffected by the airway size, as the deviations between the individuals are comparatively small. In contrary, for the younger subjects, a notable higher amount of aerosols deposits in the tracheobronchial tree, see Fig. 7c, achieving an overall higher deposition rate. We relate a higher deposition in the upper airways (mouth-throat region and tracheobronchial tree) to a lower amount of aerosols that reach the collectors that represent the alveolar region. Therefore there are less contagious particles that could reach the alveolar region of the lung, as highlighted in Fig. 7d. This trend continues for $d_p=10\,\upmu$ m aerosols, where the highest aerosol deposition is observed. For this diameter the youngest individual (Age 1) deposits approximately $~90\%$ of inserted aerosols, resulting in the lowest amount of aerosols and thus virus load that travel further into the collectors, which represent the smaller airway regions. For all considered age groups and diameters, the lung size has a major impact on the deposition effect in the tracheobronchial tree, whereas the mouth-throat region remains mainly unaffected. As stated in [5] there is a close correlation of SARS-CoV-2 viral load in the lower airways and severity in COVID-19 ARDS. In this context, we conjecture that the lower aerosol and consequently lower virus dose in the alveolar region among the children models is related to an increase of the probability of a mild infection of COVID-19. Furthermore, by increasing the age of the subject and thus the lung volume, the deposition fractions approach the results of the adult reference case.

**Fig. 8:**
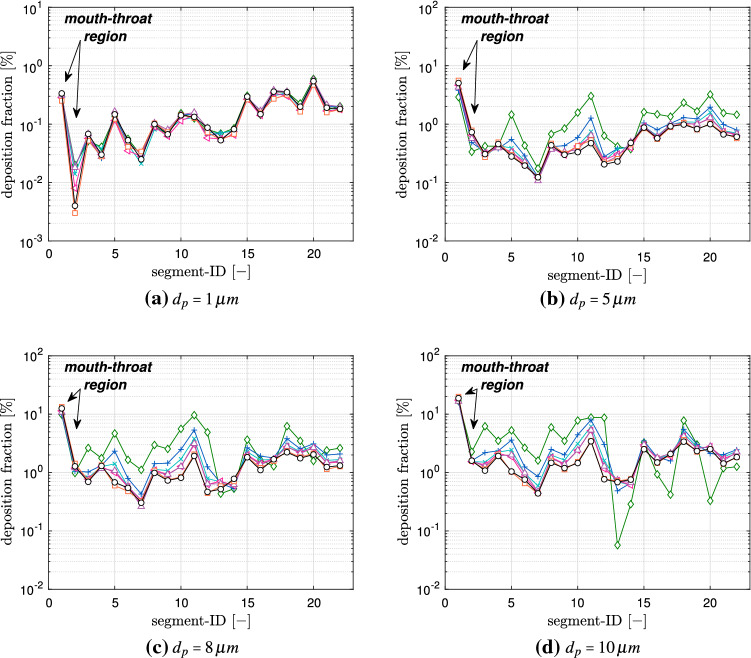
Segmental aerosol deposition for different lung sizes;  Child (Age 1),  Child (Age 3),  Child (Age 5),  Child (Age 7),  Child (Age 9),  Child (Age 13),  Adult (Male).. (Color figure online)

**Fig. 9:**
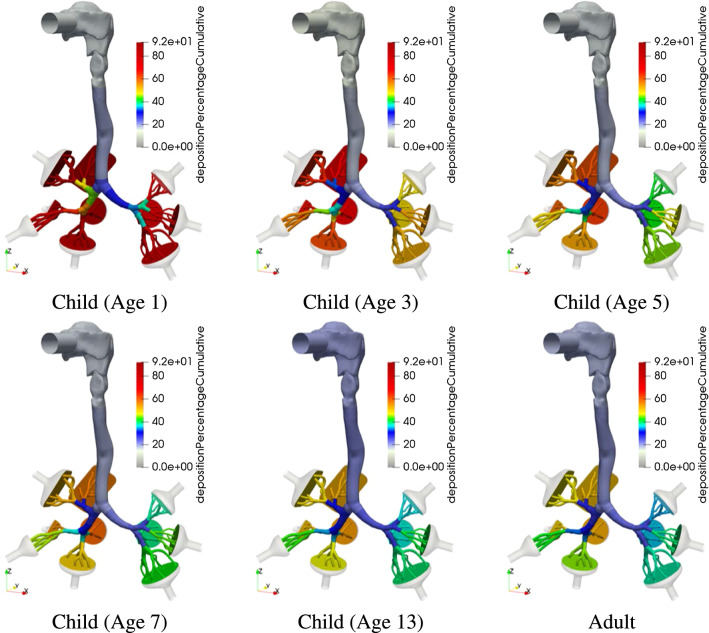
Cumulative aerosol deposition percentage along airway branches for various age-groups ($d_p=10\,\upmu$ m)

A more detailed view of the local deposition is provided in Fig. 8, where the aerosol deposition fraction is displayed over each lung model component. The allocation of these segment identifiers (segment-ID) to the lung model is provided in Fig. 1c. The component-wise deposition fractions in Fig. 8 underline the effect that the particle deposition fractions of different diameters in the mouth-throat region are nearly identical for all age groups. In addition, Fig. 8b–d indicates the correlation between an increasing deposition effect of the upper airways and a reduced airway dimension. The segmental deposition is nearly identical for all considered age groups, for particles with a diameter of $d_p=1\,\upmu$ m. The aerosols with a diameter $d_p=10\, \upmu$ m are more prone to deposit in the upper airway regions. For the child (Age 1) that is inhaling aerosols with a diameter of $d_p=10\,\upmu$ m most of the particles deposit in the first 13 segments. This effect is further visualized in Fig. 9 which presents the cumulative aerosol deposition fraction along each airway branch for $d_p=10\,\upmu$ m across six age-groups. The cumulative aerosol deposition fraction along each airway branch was calculated by summing fractional deposition in the current and all preceding segments. It is highlighted that the younger the considered individual the higher the cumulative deposition fraction in upper airway generations. Due to this increase, the deposition fraction in the lower airway regions is significantly reduced and less aerosols reach the collectors. This effect is mitigated if smaller particles are considered as presented in Fig. 8. Furthermore Fig. 8 displays that the deposition increases with larger aerosol diameter for all individuals. In all cases, the mouth-throat region has a high deposition rate, however, a difference between different age groups becomes clear in the tracheobronchial tree for the particles with $d\ge 5 \,\upmu$ m, as with the decreasing of the age of the individual and consequently the lung size, the particle deposition significantly increases.

In our study we relate the volume of air that is breathed in and out in a single breath (tidal volume *TV*) in resting condition to the estimated weight of the considered child, see Eq. 14. This leads to a *TV* of $72\,\mathrm{ml}$ for the child (Age 1) compared to $TV=630\,\mathrm{ml}$ for adults. The breathing frequency of these age-groups are estimated as $f_{Age 1}=32\,\mathrm{breaths/min}$ and $f_{Adult}=12\,\mathrm{breaths/min}$ respectively. Compared to the adult a child has therefore a higher breathing frequency as well as a reduced tidal volume. We observe, that the higher the frequency-volume ratios the higher the deposition in the upper airways. This consequently leads to a significant reduction of aerosols that can penetrate deeper into the lung. These findings relate exclusively to person’s normal activity, excluding exercise activity. More details on the average *TV* and breathing frequencies during the active phase as well as the regeneration phase of physical activities would be needed to assess the safety of exercising.

#### Time dependent deposition

The time dependent deposition of aerosol particles with $d_p=1,\,\,5,\,\,10\,\upmu$ m is compared across seven different airway dimensions. The aim is to estimate the percentage of deposited particles after one inhalation. To this end, the mean inhalation time is estimated as half of the inverse of the breathing frequency:

15 $$\begin{aligned} t_{inh}=\frac{1}{2f_{avg}}. \end{aligned}$$

The average breathing frequency $f_{avg}$ and the estimated mean inhalation time $t_{inh}$ are provided in Table 5.

**Table 5:** Average breathing frequency $f_{avg}$ and inhalation time $t_{inh}$ [27]

Child	Age 1	Age 3	Age 5	Age 7	Age 9	Age 13	Adult
$f_{avg}$ [breaths/min]	32	28	28	24	24	14	12
$t_{inh}$ [s]	0.94	1.07	1.07	1.25	1.25	2.14	2.50

**Fig. 10:**
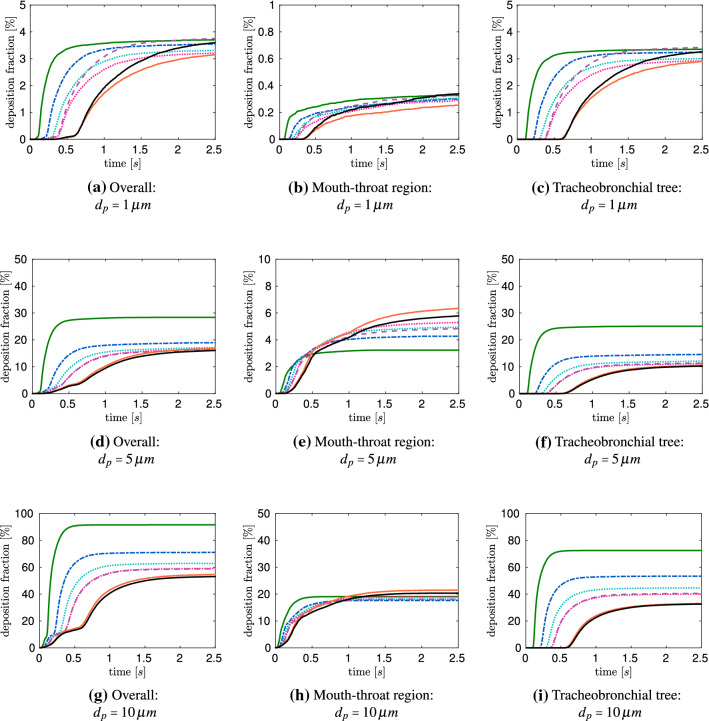
Time dependent aerosol deposition for different lung sizes:  Child (Age 1),  Child (Age 3),  Child (Age 5),  Child (Age 7),   Child (Age 9),  Child (Age 13),  Adult (Male). (Color figure online)

Figure 10 displays the resulting deposition fractions over a timespan of $2.5\,$s. Figure 10a–c represents the deposition behavior for $d_p=1\,\upmu$ m aerosols. It underlines the findings of Sect. 4.3.1, which state that the considered age groups reach a similar stationary deposition fraction in the mouth-throat as well as the tracheobronchial tree for small aerosols with $d_p=1\,\upmu$ m. Moreover, Fig. 10a–c indicates a different time dependent deposition. Due to the higher flow rates inside the smaller lungs, the particles tend to reach their final deposition state earlier. Figure 10d–f presents the time dependent deposition for $d_p=5\,\upmu$ m aerosols. For this aerosol dimension, a significant increase of the deposition rate is observed for the mouth-throat as well as the tracheobronchial tree, reaching an overall filtering of approximately $20{-}30\,\%$. It is shown that the lung size impacts the deposition rate as well as the time dependent deposition. In Fig. 10a–i it is visible that all particles are already deposited after the time needed for one inhalation (Table 5), this indicates, that all non-deposited particles reach the collectors.

#### 15 min: Inhalation

In the final step, the deposited aerosols after $15\,min$-inhalation are investigated. This time span was chosen, according to the recommendations of the German Health Departments, which predict a high risk, if an individual had distinct cumulative face-to-face contact with a host for at least $15\,min$ [29].

These results are obtained by taking the particle deposition fractions presented in Sect. 4.3.1 times the inhaled aerosols for each subject after $15\,$min. To estimate the aerosol load after $15\,$min, we assumed that an infected individual released contagious aerosol by a single cough in the inhalation region ($1\,\mathrm{m}\times 1\,\mathrm{m} \times 1\,\mathrm{m}$) of the subject. Lindsley et al. [22] measured 900–302,200 *particles*/*cough* while subjects had influenza. Therefore, an average aerosol load in a cubic room of $l_{aerosols}=150,000/\mathrm{m}^3$ is considered. The volume that the specific subjects inhales in the critical time $Q_{15min}$ is provided in Table 6. The equation for the inhalation load of aerosols $n_{15min}$ after $15\,min$ renders

16 $$\begin{aligned} n_{15min}= l_{aerosols} \times Q_{15min}. \end{aligned}$$

**Table 6:** Information for 15 min inhalation

Child	Age 1	Age 3	Age 5	Age 7	Age 9	Age 13	Adult
$Q_{inlet}$ [$\mathrm{l/min}$]	2.30	3.17	3.53	3.87	4.94	4.53	6.26
$Q_{15 \mathrm{min}}$ [$\mathrm{l}/15 \mathrm{min}$]	34.56	47.52	52.92	58.10	74.09	67.91	93.96
$n_{15 \mathrm{min}}^{\mathrm{a}}$ [$aerosols/15\,\mathrm{min}$]	5,184	7,128	7,938	8716	11,113	10,187	14,094
$n_{15 \mathrm{min}}/n_{15min}(adult)$ [$\%$]	36.78	50,57	56,32	61.84	78.85	72.23	100

$$^{\mathrm{a}}$$ $$l_{aerosols}= 150,000/\mathrm{m}^3$$

**Fig. 11:**
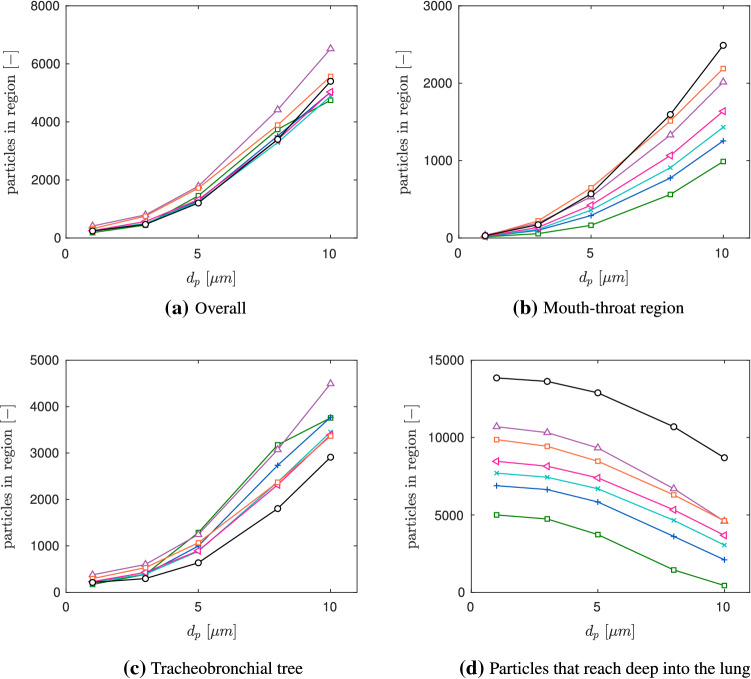
Aerosol deposition after $15\,min$-inhalation for different lung sizes;  Child (Age 1),  Child (Age 3),  Child (Age 5),  Child (Age 7),  Child (Age 9),  Child (Age 13),  Adult (Male). (Color figure online)

Table 6 shows that $n_{15 \mathrm{min}}$, the total amount of inhaled aerosols after $15\,\mathrm{min}$, varies significantly across the considered age-stages. The inhalation dose is strongly increasing with age and leads for the adult to approximately one third more aerosols compared to the adolescent (Age 13) and almost three times more aerosols compared to the youngest individual compared to the adult model. Figure 11a–d presents the deposited particles over the particle diameter for the seven different lung sizes. Moreover, Fig. 11d shows that the trend of Sect. 4.3.1 is more pronounced. This is due to the effect of increased deposition in the upper airway for younger individual and the likewise reduction of inhalation aerosol load. For the child (Age 1) the aerosol load that reached into the collectors and therefore penetrate deeper into the lung is less than one third for aerosols with $d_p=1\,\upmu$ m and less than one tenth for $d_p=10\,\upmu$ m. For older children models the difference is mitigated, but remains notable.

## Conclusions

The simulation of regional aerosol deposition in human airways of different sizes, corresponding to different age groups, is an important step to gather knowledge about the unequal distribution of COVID-19 infection, especially among younger age groups, also from a fluid mechanical point of view. In order to conduct this research, one of the current limitations was the lack of detailed lung models for different age groups. To solve the problem the considered lung geometries were generated by a dedicated scaling of the adult lung of Koullapsis et al. [18], and then used in computational models to study general trends in deposition of aerosols. The generated lung models covered artificial models of children aged $1{-}13$ years as well as the adult model of Koullapsis et al. [18]. The computational model is based on RANS equations with k-$\omega$-SST model employed to account for the turbulent flow in the airways. With the Lagrangian particle tracking the *StochasticRandomRAS* model of OpenFOAM$^{\circledR }$ is used to account for the impact of turbulent eddies on the particle trajectories. The benchmark LES1 case of Koullapsis et al. [18] served for verification of the implemented computational model. The present model leads to computational results with reasonably small differences to the reference, rendering it as suitable for investigations of the impact of different lung sizes on aerosol deposition. Aerosol deposition results are obtained and presented for different lung sizes and particle dimensions. Significant variability in regional aerosol deposition is observed across the considered airway models. However, the differences mitigated towards the smaller aerosols. In this context, deposition was found to be particularly sensitive in the tracheobronchial tree and less impacted in the mouth-throat region among the considered age groups. In addition, a general trend is observed, which indicated a higher deposition of aerosols in the upper airways for younger individuals and therefore a reduced deposition in the lower airways. A higher alveolar virus load in the lower airways is conjectured to trigger lower respiratory tract symptoms, like pneumonia or acute respiratory distress syndrome, which are also known to increase morbidity of COVID-19 patients [5, 33].

We conclude that a higher aerosol deposition in the upper airways of children, mainly in the tracheobronchial tree, leads to a significant reduction of virus load in the lower airways. We connect this effect to a higher chance of developing mild to moderate respiratory illness. Regarding our results, we propose a possible relation between the age related mechanical protection of the lung, due to airway size, and the risk of severe respiratory illness originating from COVID-19 airborne transmission. The presented computational study is applicable also to a more complete upper airway geometry case, that would also include the nasal cavity, with possibility to study also other inhalation regimes.

## Footnotes

1 In few isolated cases, where the particle tracking of OpenFOAM-V7 fails, we deactivate the particle causing the issue. This is a known issue, which is already being addressed at OpenFOAM Issue Tracking (ID: 2964). We consider this action as justified as we neglect at most one particle.
